# Alcohol as a Novel Trigger for Cannabis Hyperemesis Syndrome

**DOI:** 10.7759/cureus.102439

**Published:** 2026-01-27

**Authors:** Umair Hayat, Mohammed K Al-Sawalha, Shahzad Hussain

**Affiliations:** 1 Internal Medicine, Lady Reading Hospital Peshawar, Peshawar, PAK; 2 Internal Medicine, Jordan University Hospital, Amman, JOR; 3 Pulmonary and Critical Care, MedStar Union Memorial Hospital, Baltimore, USA

**Keywords:** alcohol, cannabis hyperemesis syndrome, case report, disease triggers, substance-related disorders

## Abstract

Cannabis hyperemesis syndrome (CHS) is a paradoxical condition occurring in chronic cannabis users, characterized by cyclic nausea, vomiting, and abdominal pain. While the primary trigger is cannabis itself, other precipitants remain poorly defined. We present the case of a 52-year-old male with recurrent CHS who experienced five distinct hyperemetic episodes, each occurring approximately one week after ingesting a single dose of alcohol. His most recent presentation was complicated by severe, life-threatening hyponatremia requiring intensive care unit management. Diagnostic workup confirmed CHS and excluded other pathologies. The consistent temporal pattern observed across multiple episodes suggests that a single dose of alcohol may be a novel and specific trigger for CHS. This case highlights a previously underreported precipitant and underscores the syndrome's potential for severe metabolic complications. Clinicians should consider inquiring about alcohol use in patients with recurrent CHS, as its identification could be pivotal for prevention strategies and patient counseling.

## Introduction

Cannabis hyperemesis syndrome (CHS) is a clinical disorder characterized by a triad of symptoms: cyclic nausea, profuse vomiting, and abdominal pain, which paradoxically manifests in individuals with a history of chronic, heavy cannabis use [1]. Diagnosis of CHS is clinical and based on characteristic features, including chronic or frequent cannabis use, recurrent episodes of severe cyclic nausea and vomiting, abdominal pain, relief of symptoms with hot showers or baths, and resolution of symptoms with sustained cannabis cessation. Supportive features include weekly cannabis use and younger age, with alternative causes of vomiting excluded based on clinical assessment and investigations. [2] As cannabis use continues to rise among various age groups, particularly for recreational purposes among adolescents, it is also being increasingly used for medical reasons. This includes its application as an antiemetic for chemotherapy-induced vomiting, an appetite stimulant for individuals experiencing cachexia, and as an analgesic for conditions like peripheral neuropathies. Additionally, cannabis is used as an antispasmodic treatment for multiple sclerosis [3]. Given this growing trend, it is important to identify and understand the triggers that can lead to CHS.

The pathophysiological mechanism of CHS is believed to stem from dysregulation of the endocannabinoid system (ECS). Chronic and heavy exposure to tetrahydrocannabinol (THC), a potent cannabinoid receptor 1 (CB1) agonist, is thought to lead to complex neuroadaptations, including receptor downregulation and functional antagonism in key regions of the central nervous system [4]. The hypothalamus, which regulates body temperature and nausea, is a primary site of this dysregulation, explaining the thermoregulatory compulsions and cyclic vomiting [5]. Furthermore, CB1 receptor dysfunction in the gastrointestinal tract can disrupt normal gastric motility and peristalsis, contributing to abdominal pain and nausea [4].

The primary trigger for CHS episodes is the use of cannabis itself, yet the potential for other substances to precipitate attacks is poorly understood. Alcohol consumption presents a particularly compelling and under-investigated candidate, given its widespread use. Moreover, the co-use of cannabis and alcohol is highly prevalent in the general population, increasing the likelihood of their interactive effects being observed clinically [6].

Despite the plausible biological connection, the role of alcohol as a specific precipitant of CHS episodes remains poorly documented in the medical literature. Current evidence is largely confined to mechanistic studies, with a notable scarcity of clinical case reports that establish a clear, temporal link. We present a case of a 52-year-old male with recurrent CHS who experienced five distinct hyperemetic episodes, each predictably occurring after a single dose of alcohol, highlighting a potential and previously underreported trigger that warrants greater clinical awareness.

## Case presentation

A 52-year-old male presented to the emergency department with a four-day history of severe nausea, profuse vomiting (four to five episodes per day), diffuse abdominal pain rated eight out of 10 in severity, and associated lightheadedness, lower limb weakness, and loose stools. He reported significant symptomatic relief with hot showers. His history was significant for chronic cannabis use (approximately 2 grams daily for over 30 years), daily cigar use, and a prior history of heavy alcohol consumption that he had reduced to social drinking over a year prior to presentation. At the time of presentation, he was not taking any prescription or over-the-counter medications apart from intermittent oral ondansetron for nausea. Notably, he was not using diuretics, selective serotonin reuptake inhibitors, antipsychotics, or glucagon-like peptide-1 receptor agonists.

The patient identified a consistent precipitant for his current and previous hyperemesis episodes: the ingestion of a single shot of alcohol (approximately 44 mL) approximately one week prior to symptom onset. He recalled this same temporal pattern preceding four prior presentations for cannabis hyperemesis syndrome (CHS) in June 2021, February 2023, January 2025, and one additional undocumented episode. Due to poor oral intake from vomiting, he reported decreased fluid intake and associated weight loss.

The patient's history was significant for recurrent hospitalizations for CHS, consistently complicated by profound hyponatremia, establishing a clear pattern of susceptibility. His first documented episode in 2021 was marked by a critically low sodium level of 98 mmol/L. This pattern continued with a presentation in February 2023, where he was discharged with a sodium level of 122 mmol/L after brief management, and another episode in January 2025, with a discharge sodium of 131 mmol/L. He denied the use of other substances, recent illness, or medications that could explain these recurrent electrolyte disturbances.

Upon admission, the patient was hemodynamically stable, with vital signs within normal physiological parameters, as detailed in Table 1.

**Table 1:** Vital signs on admission. This table summarizes the patient's vital signs upon presentation to the emergency department. The values were measured using standard, non-invasive clinical techniques: tympanic thermometry for temperature, automated sphygmomanometer for blood pressure and heart rate, and pulse oximetry for oxygen saturation. All recorded parameters were within normal physiological ranges, indicating hemodynamic stability and the absence of fever or hypoxia at the time of assessment.

Parameter	Patient Value	Normal Range	Impression
Temperature	36.8°C	36.1 - 37.2°C	Afebrile
Heart Rate	77 beats per minute	60 - 100 beats per minute	Normal
Blood Pressure	116/83 mmHg	<120/80 mmHg	Normal
Respiratory Rate	16 breaths per minute	12 - 20 breaths per minute	Normal
Oxygen Saturation	97% on room air	>95%	Normal

On physical examination, he exhibited signs of dehydration, including dry oral mucosa, but had normal skin turgor. Abdominal examination revealed a soft, non-tender, and non-distended abdomen. Neurological examination was intact; he was alert and oriented to person, place, and time. 

The initial laboratory evaluation, however, revealed significant metabolic abnormalities indicative of severe volume depletion and electrolyte loss, all of which are comprehensively outlined in Table 2. Notable diagnostic results included a urine toxicology screen positive only for cannabis.

**Table 2:** Pertinent laboratory findings on admission. This table presents key laboratory results from blood and urine samples obtained at admission. The serum studies, analyzed via standardized automated clinical chemistry analyzers, reveal a profound hypotonic hyponatremia with hypochloremia and metabolic alkalosis, consistent with volume depletion from vomiting. The low urine sodium and chloride levels support a diagnosis of hypovolemic hyponatremia. The leukocytosis is likely secondary to hemoconcentration. The toxicology panel, performed using enzyme immunoassay, was positive only for cannabinoids, confirming recent cannabis use while ruling out other common substances or acute alcohol intoxication as contributing factors.

Test	Patient Value	Reference Range	Impression
Serum Studies			
Sodium	117 mmol/L	136-145 mmol/L	Severe hyponatremia
Chloride	77 mmol/L	98-107 mmol/L	Hypochloremia
Bicarbonate	34 mmol/L	22-29 mmol/L	Metabolic alkalosis
Serum Osmolality	259 mOsm/kg	275-295 mOsm/kg	Hypotonicity
Serum Uric Acid	5.6 mg/dL	3.5–7.2 mg/dL	Normal
Urine Studies			
Urine Sodium	11 mmol/L	>20 mmol/L	Low, consistent with volume depletion
Urine Chloride	<20 mmol/L	>20 mmol/L	Low, consistent with volume depletion
Urine Osmolality	98 mOsm/kg	300–900 mOsm/kg	Inappropriately dilute urine, suggesting inadequate ADH response despite volume depletion
Toxicology			
Ethanol Level	<3 mg/dL	<10 mg/dL	Negative
Urine Drug Screen	Positive for Cannabis	Negative	Confirms recent use

Importantly, metabolic and endocrine contributors to emesis and hyponatremia, including dysglycemia, hypothyroidism, and adrenal insufficiency, were excluded. In contrast, hyperlipidemia was identified as a chronic comorbidity and managed with high-intensity statin therapy, as summarized in Table 3.

**Table 3:** Metabolic, Endocrine, and Lipid Profile Evaluation Normal thyroid, adrenal, and glucose parameters helped exclude metabolic and endocrine etiologies for recurrent vomiting and hyponatremia.

Test Category	Parameter	Patient Value	Reference Range	Interpretation
Lipid Profile	Total Cholesterol	242 mg/dL	<200 mg/dL	Elevated
	LDL Cholesterol	168 mg/dL	<100 mg/dL	Elevated
	HDL Cholesterol	38 mg/dL	>40 mg/dL	Low
	Triglycerides	214 mg/dL	<150 mg/dL	Elevated
Glucose Metabolism	Fasting Blood Glucose	96 mg/dL	70–99 mg/dL	Normal
Endocrine Evaluation	Thyroid-Stimulating Hormone (TSH)	3.03 μIU/mL	0.4–4.5 μIU/mL	Normal
	Morning Serum Cortisol	15.9 μg/dL	6–23 μg/dL	Normal

**Figure 1:**
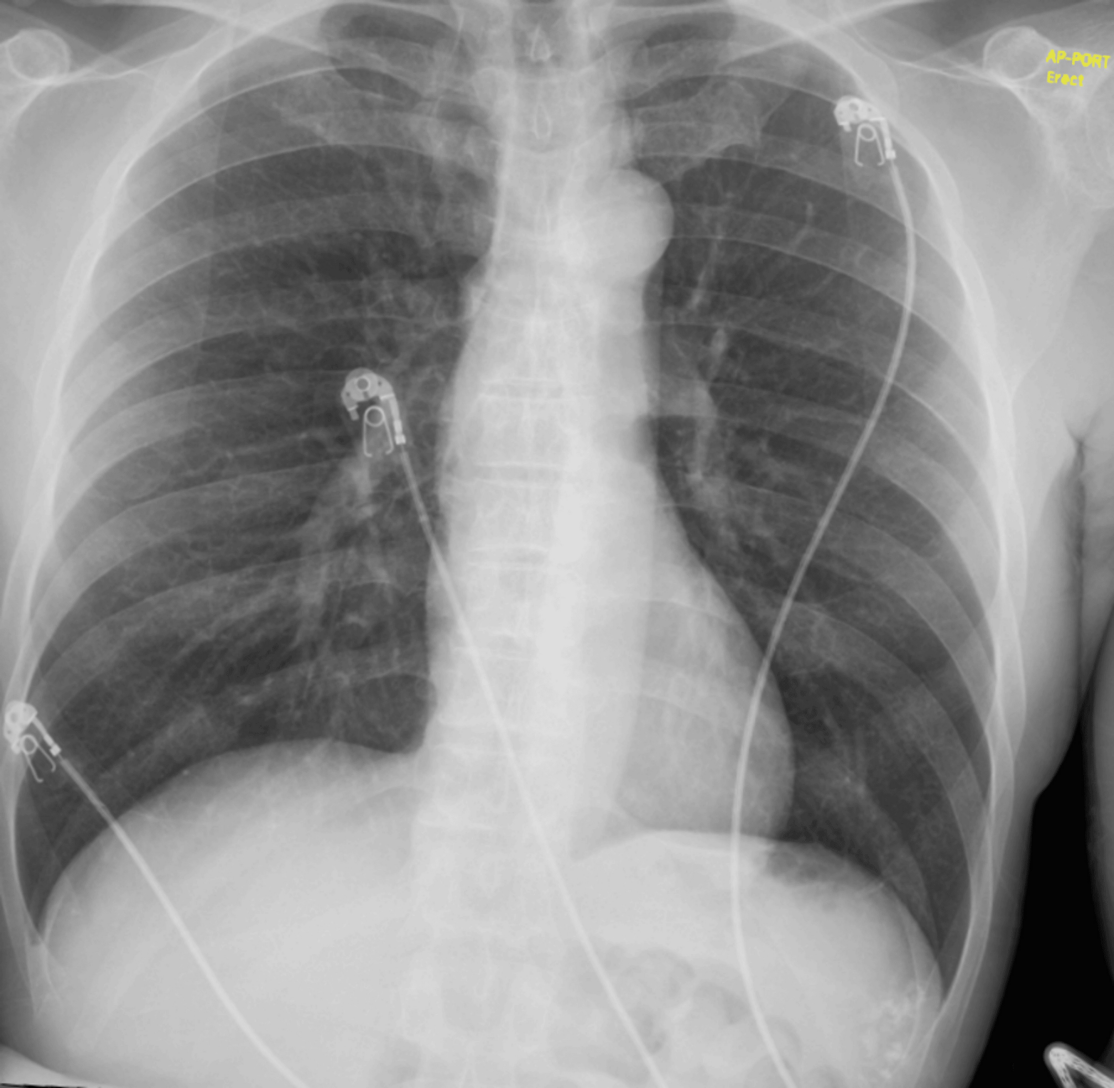
Normal chest radiograph obtained during admission Chest radiograph obtained on admission was unremarkable, with no acute cardiopulmonary abnormalities.

A chest X-ray was unremarkable, as shown in Figure 1. A computed tomography (CT) scan of the abdomen from a previous admission in January 2025 showed a moderate stool burden but no evidence of acute abdominal pathology.

The patient was admitted to the intensive care unit for management of severe hyponatremia. He was treated with a 500 mL normal saline bolus followed by a 0.9% saline infusion at 100 mL/hour, with a goal sodium correction rate not exceeding 4-6 mmol/L per 24 hours. Symptomatic management included intravenous ondansetron (4 mg every four hours as needed) and famotidine (20 mg IV).

His clinical course was notable for the resolution of vomiting and stabilization of his symptoms. His sodium level was corrected to 131 mmol/L over a period of three days. The leading diagnosis was recurrent CHS precipitating a hypovolemic, hypotonic hyponatremia due to profound vomiting. The observed leukocytosis was attributed to hemoconcentration. Further abdominal imaging was deferred due to the recent normal CT scan and the absence of signs suggesting an acute surgical or infectious process. The patient was offered cannabis cessation counseling and nicotine replacement therapy, but declined both upon discharge.

## Discussion

CHS represents a paradoxical and clinically challenging condition that manifests in long-term cannabis users, characterized by cyclic episodes of severe nausea, vomiting, and abdominal pain, often accompanied by the pathognomonic compulsion to take hot showers [1]. Our case of a 52-year-old male with recurrent, severe CHS episodes complicated by hyponatremia offers a critical opportunity to explore the syndrome's diagnostic intricacies, its profound metabolic consequences, and, most notably, introduces a potentially novel precipitant: a single dose of alcohol.

The diagnosis of CHS rests upon a triad of features: chronic cannabis use, stereotypical symptomatology, and the exclusion of other causes [7]. Our patient fulfilled these criteria, with a 30-year history of heavy cannabis use confirmed by toxicology, a classic presentation with compulsive hot bathing providing relief, and an unremarkable abdominal CT scan ruling out surgical pathology. Importantly, alternative iatrogenic causes of nausea, vomiting, and hyponatremia were carefully excluded. The patient was not taking diuretics, selective serotonin reuptake inhibitors, antipsychotics, or glucagon-like peptide-1 receptor agonists, all of which have been independently associated with gastrointestinal intolerance or electrolyte disturbances. The absence of these potential pharmacologic confounders strengthens the attribution of both the recurrent emesis and resultant hypovolemic hyponatremia to cannabis hyperemesis syndrome and its associated behaviors, rather than medication-induced pathology. The primary challenge in diagnosis often lies in patient self-awareness and clinician suspicion, as many patients are unaware of the syndrome and may not volunteer their cannabis use history without direct questioning. This case underscores the necessity of routine and non-judgmental substance use screening in patients presenting with refractory vomiting.

A central and novel aspect of our case is the consistent temporal relationship between ingestion of a single dose of alcohol and the onset of hyperemesis approximately one week later, observed across five distinct episodes. This delay argues strongly against acute alcohol toxicity or withdrawal and instead supports a threshold-based mechanism in which alcohol acts as a destabilizing trigger within a chronically dysregulated endocannabinoid system. Chronic cannabis use is associated with CB1 receptor downregulation and impaired gut-brain axis signaling, creating a fragile compensatory state that normally suppresses emesis. Preclinical evidence indicates that alcohol exposure can alter endocannabinoid signaling, including changes in endocannabinoid levels and CB1 receptor expression in both central and peripheral tissues, effects that may persist beyond acute intoxication [8]. In a susceptible individual, a single alcohol exposure may gradually erode compensatory control, culminating in delayed hyperemesis once regulatory mechanisms fail [3]. The reproducibility of this delayed pattern across multiple episodes strengthens the plausibility of alcohol as a specific precipitant rather than a coincidental exposure. Nevertheless, the precise neurobiological mechanisms underlying this interaction remain incompletely understood and warrant further mechanistic and clinical investigation.

The most life-threatening complication in our patient was severe hyponatremia, with a sodium level of 117 mmol/L. The hyponatremia was determined to be hypovolemic hypotonic hyponatremia secondary to excessive vomiting and free-water intake in the setting of cannabis hyperemesis syndrome. He was not receiving diuretics, and endocrine causes were excluded, with a normal thyroid-stimulating hormone level of 3.03 μIU/mL and a normal morning cortisol level of 15.9 μg/dL. Urine studies demonstrated low urine sodium (11 mmol/L), low urine chloride (<20 mmol/L), and low urine osmolality (98 mOsm/kg), consistent with extrarenal sodium loss and appropriate renal sodium conservation in response to volume depletion. The low serum osmolality of 259 mOsm/kg further supports a hypotonic etiology. Although hypovolemia typically stimulates antidiuretic hormone release, the low urine osmolality observed may reflect a non-steady-state condition following recent intravenous fluid administration, resulting in transient suppression of ADH activity. Despite this, urine sodium and chloride levels remained low, consistent with persistent activation of the renin-angiotensin-aldosterone system.

Importantly, the clinical chronology supports vomiting as the primary driver of hyponatremia rather than the reverse. The patient experienced four days of persistent emesis and poor oral intake while consuming large quantities of free water before presentation. His nausea and vomiting improved rapidly with antiemetic therapy and isotonic fluid resuscitation, preceding complete correction of serum sodium levels. Throughout hospitalization, his mentation remained intact, without headache, seizures, or altered consciousness, which would typically be expected if hyponatremia were the primary cause of vomiting. The patient was not receiving beta-blockers or other rate-controlling medications. Despite volume depletion, he remained hemodynamically stable, with preserved blood pressure and no orthostatic symptoms, suggesting a compensated hypovolemic state. Therefore, the absence of tachycardia does not exclude hypovolemic hypotonic hyponatremia. Furthermore, although the patient’s episodes followed alcohol ingestion, his serum ethanol was negligible (<3 mg/dL), and he did not engage in recent heavy or binge drinking. Laboratory and dietary evaluation exclude low-solute alcohol intake (beer potomania) as a cause of hyponatremia, supporting vomiting-induced, hypovolemic hypotonic hyponatremia in the context of CHS, with alcohol acting as a reproducible trigger rather than a direct metabolic contributor.

Hyponatremia is a recognized but potentially life-threatening complication of CHS, resulting from profound vomiting and replacement of losses with hypotonic fluids such as water. The laboratory profile characterized by low urine sodium, low urine osmolality, and metabolic alkalosis is indicative of extrarenal sodium loss and volume depletion [9]. This case highlights that CHS is not a benign condition and may lead to severe metabolic derangements requiring intensive care management. Correction of profound hyponatremia must be approached cautiously to avoid catastrophic complications such as osmotic demyelination syndrome [10].

Finally, the long-term management of CHS remains its most significant hurdle, centered almost exclusively on cannabis cessation [11]. Our patient's repeated refusal of cessation counseling underscores a common and frustrating theme in the clinical course of CHS. Many patients are either reluctant to believe cannabis is the cause, given its antiemetic properties in acute use, or are unable to discontinue use due to dependence [12]. This highlights the critical need for multidisciplinary approaches involving addiction specialists and persistent, empathetic patient education at every clinical encounter to break the cycle of recurrence.

In conclusion, this case reinforces the diagnostic criteria and serious complications of CHS while proposing alcohol as a novel and specific trigger. The precise temporal pattern observed suggests a unique drug-drug interaction worthy of further pharmacological and clinical investigation. Clinicians should be aware of this potential trigger and maintain a high index of suspicion for CHS in any chronic cannabis user presenting with unexplained cyclic vomiting, ensuring prompt diagnosis and aggressive management of its metabolic sequelae.

## Conclusions

This case provides critical clinical insights by identifying a single dose of alcohol as a potential novel trigger for CHS, expanding the known spectrum of precipitants. It underscores the serious morbidity of CHS, which can progress to life-threatening complications like severe hyponatremia necessitating intensive care. Furthermore, it highlights the persistent challenges in management, including patient reluctance to accept the diagnosis and the difficulty of achieving sustained cannabis cessation. Ultimately, this report suggests that directly inquiring about alcohol consumption should be integrated into the clinical evaluation of patients with recurrent CHS, as identifying this trigger could be pivotal for prevention and effective patient counseling.
